# Antidiabetic Properties of the Root Extracts of Dandelion (*Taraxacum officinale*) and Burdock (*Arctium lappa*)

**DOI:** 10.3390/plants13071021

**Published:** 2024-04-03

**Authors:** Daria Zolotova, Renāte Teterovska, Dace Bandere, Liga Lauberte, Santa Niedra

**Affiliations:** 1 Department of Pharmaceutical Chemistry, Faculty of Pharmacy, Rīga Stradiņš University, LV-1007 Riga, Latvia; renate.teterovska@rcmc.lv (R.T.); dace.bandere@rsu.lv (D.B.); 2 Department of Pharmaceuticals, Red Cross Medical College, Rīga Stradiņš University, LV-1007 Riga, Latvia; 3 Baltic Biomaterials Centre of Excellence, Headquarters at Riga Technical University, LV-1658 Riga, Latvia; 4 Laboratory of Finished Dosage Forms, Riga Stradiņš University, LV-1007 Riga, Latvia; liga.lauberte@rsu.lv (L.L.); santa.niedra@rsu.lv (S.N.)

**Keywords:** diabetes mellitus, dandelion, burdock, tannin, inulin, total polysaccharide, total phenolic content, antioxidant, hypoglycemic properties

## Abstract

Several preclinical studies suggest the potential of edible plants in controlling blood sugar levels and stabilizing diet. The goals of the study were to examine, analyze, and describe whether there are chemical compounds in dandelion and burdock roots that could have antidiabetic properties. The 70% ethyl alcohol and lyophilizate extracts (AE and LE, respectively), were used, and analyses were carried out on their total polysaccharide (TP), total phenolic content (TPC), tannin, and inulin. The antioxidant activity of extracts was determined using the DPPH (2,2-diphenyl-1-picrylhydrazyl) assay, and hypoglycemic properties were based on α-amylase activity. Liquid chromatography–mass spectrometry was used for the tentative identification of the chemical components. Qualitative techniques confirmed the presence of inulin in both roots. Analysis of TPC, tannin content, DPPH assay, and α-amylase activity revealed higher values for burdock compared to dandelion. However, dandelion exhibited higher TP content. Burdock contained a small amount of tannin, whereas the tannin content in dandelion was insignificant. All LE consistently exhibited higher values in all analyses and assays for all roots compared to AE. Despite burdock root showing overall better results, it is uncertain whether these plants can be recommended as antidiabetic agents without in vivo studies.

## 1. Introduction

Diabetes mellitus (DM) is a chronic noninfectious disease that is spread worldwide, categorized into three main types: type 1 DM (T1DM), type 2 DM (T2DM), and gestational DM (GDM) [1]. The International Diabetes Federation [2] reports that around 540 million people develop diabetes, with T2DM accounting for 90% of the cases [3]. T2DM is one of the main chronic diseases and a serious long-term metabolic disorder, which develops because of an imbalanced diet, insufficient physical activity, and genetic factors. This metabolic disorder results from both inadequate insulin secretion by pancreatic β-cells and diminished tissue responsiveness to insulin [4]. Hyperglycemia, a hallmark of DM [5], can lead to organ and tissue damage, contributing to complications such as cardiovascular disease, kidney issues, and eye diseases [6]. Timely administration of hypoglycemic drugs is essential for diabetics to regulate blood glucose levels and manage the risk of complications. The critical role in increasing blood sugar, and the pathogenesis and progression of DM also has oxidative stress, which results in reducing insulin production by pancreatic islets [7].

Given the drawbacks and costs associated with various chemical hypoglycemic agents used to treat DM, there is a growing trend towards herbal medicine for controlling and managing this disease [8]. Presently, consumers are increasingly attracted to natural products due to their notable efficacy and low toxicity. Several preclinical studies and reviews have demonstrated the potential effects of edible plants in controlling blood sugar levels and stabilizing diets as well [9,10,11,12].

Throughout history, plants have played a crucial role in benefiting humanity. Medicinal plants, known for their diverse chemical compounds and various biological activities, including antioxidant and hypoglycemic properties, significantly contribute to our well-being. Some of these plants renowned for their multifaceted health effects are dandelion (*Taraxacum officinale*) [13] and burdock (*Arctium lappa*) [14], both are members of the family Asteraceae or Compositae. These are traditional medicinal and edible plants, and their medical benefits have been known for centuries. These plants contain a diverse array of phytochemicals that exhibit specific biological activities, such as phenolic acids known for their antioxidant and immunostimulatory properties, coumarins demonstrating antitumor, anti-inflammatory, antimicrobial, and anticoagulant properties [15]; polysaccharides with antioxidant, antitumor, hypoglycemic, and immune regulation activities [16,17,18], as well as inulin, vitamins, and others. Some of these phytochemicals may assist in sustaining low blood glucose, preventing high blood pressure, and enhancing the body’s antioxidant system and insulin regulation [19]. Previously, plant extracts have been found to target the root causes of diseases and exhibit multiple mechanisms of action due to the combined effects of different plant compounds [20]. This versatility is beneficial for treating complex conditions like T2DM. This study was carried out on plant roots since it is rarely studied as part of those plants.

The goals of the study were to examine, analyze, and describe whether there are chemical compounds in the dandelion and burdock roots that could have antidiabetic properties by maintaining or lowering blood sugar levels or improving the body’s antioxidant capabilities. The research involved collecting plant samples from two distinct regions. The study also aimed to examine the data from both regions to discern the extent of differences in the chemical profiles and antidiabetic properties of the plants.

## 2. Results

### 2.1. Extract Analysis

#### 2.1.1. Identification of Inulin

In the analysis of burdock root, the application of 0.1 mL of a 20% thymol alcohol solution and 0.05 mL of concentrated sulfuric acid to the ground plant material revealed the presence of orange–red coloring, indicative of inulin. Similarly, in the analysis of dandelion root, the application of a 20% α-naphthol alcohol solution and concentrated sulfuric acid to the ground plant material resulted in the presence of a purple–pink color, confirming the presence of inulin. Notably, the absence of blue staining, indicating the absence of starch, was not observed in both cases when applying 0.1 mL of iodine solution to the analyzed powder.

The experimental results affirm the presence of inulin in both burdock and dandelion roots, as evidenced by the observed colors following the specified chemical analyses.

#### 2.1.2. Determination of Total Phenolic Content (TPC)

The 70% ethyl alcohol and lyophilizate (freeze-dried) extracts (AE and LE, respectively), were used and the results were compared to determine the total phenolic content (TPC) in dandelion and burdock roots. Results are shown in Table 1. The research reveals a statistically significant difference in the TPC between AE and LE for all extracts (*p*-value < 0.05, according to the Mann–Whitney U test). Conversely, when comparing two Latvian rural regions, no statistically significant difference was observed (*p*-value > 0.05, according to ANOVA). 

In a broader perspective, burdock exhibited superior results to dandelion, and the LE, when compared with the AE (of a specific plant), showed more favorable outcomes. The highest TPC was observed in the LE of burdock (BV) [100.97 ± 0.49 mg GSE/g], while the lowest was noted in the AE of dandelion (DV) [4.51 ± 0.03 mg GSE/g]. 

A statistical analysis showed a statistically significant difference (*p*-value < 0.05) between the TPC values of AE and LE, indicating variations in phenolic content between the two extraction methods.

#### 2.1.3. Determination of Tannin Level

**Figure 1:**
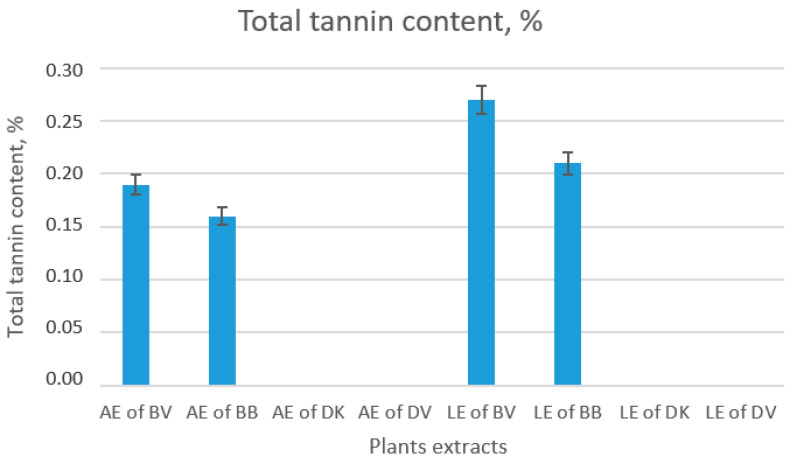
Total tannin content in dried plant extract, % (±SE). Seventy percent ethyl alcohol extract (AE), and lyophilizate extracts (LE). Burdock from “Viļani” (BV), burdock from “Būdiņas” (BB), dandelion from “Kaļķis” (DK), and dandelion from “Vecpiebalga” (DV).

The total tannin content is illustrated in Figure 1. To determine it, two types of extracts (AE and LE) were utilized. Dandelion root showed 0% tannin in both extracts, indicating a value too small to be detected. On the other hand, burdock root exhibited some value, albeit relatively low; the highest was measured in the LE of burdock (BV) with 0.27 ± 0.01%, and the lowest in the AE of BB with 0.16 ± 0.01%. The LE, when compared with the AE, demonstrated higher results (*p*-value > 0.05, according to the Mann–Whitney U test). Although the results are different for the same plant, but from different locations, this difference is not statistically significant (*p*-value > 0.05, according to ANOVA).

#### 2.1.4. Determination of Total Polysaccharide Content (TP)

**Figure 2:**
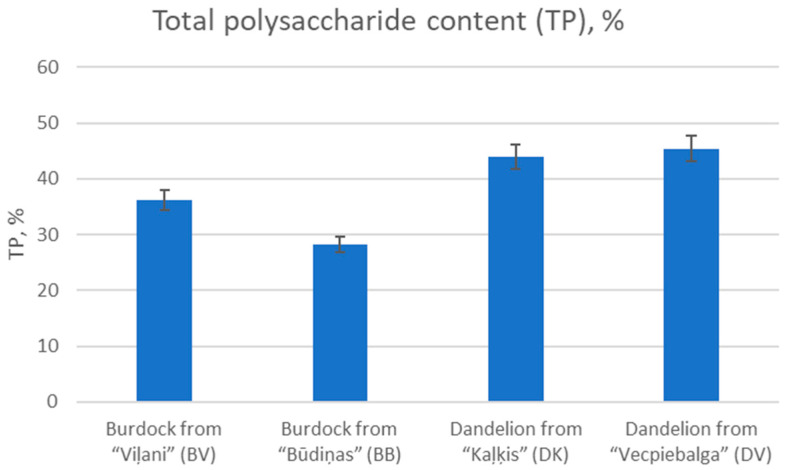
Total polysaccharide content (TP) in terms of fructose, % (± SE).

The total polysaccharide content is depicted in Figure 2. When comparing the polysaccharide content of two different plants, it is evident that dandelion root has the highest value (DK = 44.02%, DV = 45.41%), signifying a higher concentration of polysaccharides in the sample compared to burdock root (BV = 36.15%, BB = 28.22%). The research indicates non-statistically significant variations in TP between burdock and dandelion roots (*p*-value > 0.05).

### 2.2. Determination of Antioxidant Activities of Extracts by Using DPPH (2,2-diphenyl-1-picrylhydrazyl) Assay

**Figure 3:**
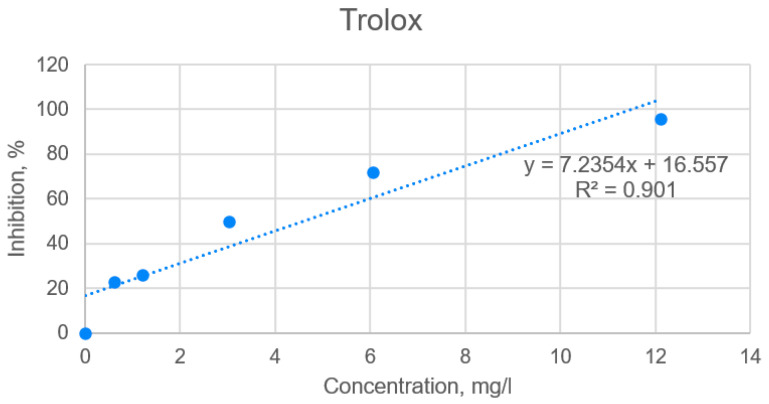
Standard curve of the Trolox.

Trolox solution was employed as a standard, and the standard curve of the Trolox solution was generated by plotting concentration against inhibition. The regression equation was expressed as follows: y = 7.2354x + 16.557, R^2^ = 0.901 (Figure 3). Before commencing the analysis of the extracts, the IC50 for Trolox was calculated, and its value equaled 4.62 ± 0.23 mg/mL.

As evident from the results in Table 2, the IC50 of the LE (IC50 from 0.77 to 9.52 mg/L) exhibited a significantly superior outcome compared to the AE (IC50 from 25.89 to 236.32 mg/L), and the difference between these two groups is statistically significant (*p*-value < 0.05, according to the Mann–Whitney U test). The LE of burdock BB demonstrated the best result, although both locations of burdock LE, when compared with Trolox, showcased greater antioxidant activity. Conversely, the AE of dandelion DK yielded the least favorable result. 

### 2.3. Hypoglycemic Properties of the Extracts Based on α-Amylase Activity

**Figure 4:**
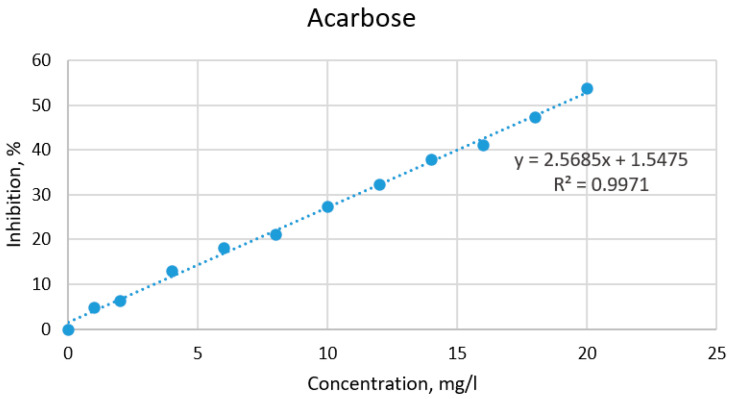
Standard curve of the acarbose.

Acarbose was utilized for the standard solution, and the standard curve was constructed by plotting concentration against inhibition using this solution. The regression equation is represented as y = 2.5685x + 1.5475, with an R^2^ value of 0.9971 (Figure 4). Prior to commencing the analysis of the extracts, the IC50 for acarbose was calculated, resulting in a value of 18.86 mg/mL.

None of the plant extracts matched or approached the value of acarbose (Table 3). The LE of burdock exhibited the most favorable outcomes (BV = 79.18 mg/mL, BB = 57.94 mg/mL), while the AE of dandelion demonstrated the least favorable results (DK = 205.35 mg/mL, DV = 450.11 mg/mL). As observed in other analyses, LE consistently exhibited higher values compared to AE within the same plant samples. The disparity between the IC50 values of the LE and AE is statistically significant (*p*-value < 0.05, according to the Mann–Whitney U test).

### 2.4. Qualitative Analysis of Extracts by Liquid Chromatography–Mass Spectrometry (LC-MS)

The qualitative analysis of extracts by LC-MS revealed numerous chemical compounds in each type of extract for all plants. Results are shown in Table 4 and Table 5. The research revealed a diverse array of chemical compounds present in the root extracts of both plants. These compounds encompassed various classes, including Amino acids, Phenolic acids, Benzoic acids, Phenolic glycosides, Alkaloids, Guaianolides, Monocyclic monoterpenoids, Oligothiophenes, Hydroxy acids, and Tannins. 

The presence of specific chemical compounds in the extracts is depicted in Table 6 and Table 7. Among the detected compounds, 3,5-dicaffeoylquinic acid, 4,5-dicaffeoylquinic acid, disaccharide, chlorogenic acid, n-caffeoylquinic acid, neochlorogenic acid, phenylalanine, valine, and arginine were present in all extracts. Additionally, caffeic acid, caffeic acid ethyl ester, oleanolic acid, p-coumaric acid, trigonelline, and arctinone A were exclusively identified in burdock samples, while salicylic acid glucoside, protocatechuic acid, campholenic aldehyde, and eremanthin were found only in dandelion extracts. These findings underscore a greater diversity of beneficial substances in burdock compared to dandelion, with burdock also exhibiting a higher number of unique chemical compounds.

## 3. Discussion

The present study explores the therapeutic potential of dandelion and burdock roots, emphasizing their chemical constituents and implications for DM and related metabolic disorders. Investigated compounds include tannins, total phenolic content, total polysaccharides, and inulin.

The chosen solvent, ethyl alcohol, is one that people can readily use, avoiding options like acetone or methanol, which might produce better results in extraction yield [21,22] but are impractical and toxic for everyday use. Additionally, we opted for lyophilization as it represents the industrial processing method and provides better storage capabilities.

Various techniques have been employed to investigate the roots. One method involved quantifying the number of polysaccharides in plants. Some studies suggested that polysaccharides possess antioxidant and hypolipidemic properties and may also contribute to lowering blood sugar levels [23,24]. The presence of inulin, a polysaccharide, which, in addition to the general properties of polysaccharides, has also been proven to regulate the flora in diabetic patients [25], was confirmed by a qualitative method. Subsequently, the total phenolic content was analyzed, encompassing tannins known for their antioxidant properties in other studies [26,27]. For the determination of antioxidant properties, the 2,2-diphenyl-1-picrylhydrazyl assay was used [28], while for hypoglycemic properties, the α-amylase activity was employed [29]. 

To reduce hyperglycemia, one approach is to slow down glucose absorption by inhibiting carbohydrate-hydrolyzing enzymes like α-amylase and α-glucosidase [30]. Therefore, our study aimed to explore how root extracts affect the activity of alpha-amylase. However, it is important to consider that diabetes is a complex condition involving various metabolic pathways [31]. So, while assessing α-amylase activity is informative, a comprehensive evaluation including factors like α-glucosidase activity [32], insulin secretion [33], and glucose uptake [34] may offer a more thorough understanding of the roots’ antidiabetic properties, specifically hypoglycemic properties. In addition, oxidative stress plays a crucial role in the development and worsening of complications of diabetes [31,35,36]. The imbalance between the production of reactive oxygen species and the body’s ability to counteract them with antioxidants leads to cellular harm and impairment. In diabetes, high blood sugar and issues with energy production in cells exacerbate this stress [37]. Therefore, the antiradical activity test was applied.

All studied plants demonstrate antiradical and α-amylase inhibitory activities. During the DPPH assay, IC50 values for the plants are compared with Trolox (an analog of vitamin E), and for α-amylase activity, with acarbose. The LE of burdock root stands out, suggesting its effectiveness in inhibiting α-amylase and preventing oxidization, while dandelion has worse results. Additional studies employing spectrometric, chromatographic, or electrochemical techniques are needed for accurate antioxidant and hypoglycemic activity assessment [38]. Carrying out DPPH and α-amylase inhibition assay, plants exhibit statistically significant differences between different locations, indicating sensitivity to environmental conditions. However, analyses conducted to identify the presence of specific chemical components in the plants separately revealed no statistically significant differences. 

Screening plant polysaccharides for antidiabetic activity has gained attention due to their health benefits and biocompatibility. Polysaccharides, natural macromolecular polymers, exhibit a range of biological activities and pharmacological effects. These include immune regulation [39], anticancer properties [40], and antioxidative effects [41]. Available research results have proven that plant polysaccharides can reduce blood glucose levels and enhance insulin sensitivity through various mechanisms [42]. Although dandelion shows fewer promising results in the current study, the difference is not statistically significant (*p*-value > 0.05). 

Inulin, a fructan-type polysaccharide carbohydrate, is known for its various health benefits such as improving nutritional values, protecting against oxidative stress, mitigating inflammation [43], and having glucose-lowering properties [44]. However, it is essential to note that inulin is not the only polysaccharide with antidiabetic properties [45,46]. There is also a study [47] where a novel polysaccharide with high antioxidant, antibacterial, and anticancer activities was isolated from dandelion. While inulin is a significant component, assessing the overall polysaccharide content offers a broader understanding of the sample’s composition and potential health benefits. Additionally, it allows for comparison with other polysaccharides present in extracts. Therefore, analyzing overall polysaccharide content enhances the comprehensiveness of the study and provides valuable insights into the potential antidiabetic properties of various polysaccharides present in the samples. Extracting inulin from plant material poses challenges due to the persistence of impurities in the final inulin powder [48]. These impurities can significantly impact the accuracy of the results, leading to inaccuracies in the analysis. Currently, efforts are focused on optimizing extraction techniques and purification processes [49] to ensure the accuracy and validity of the results obtained from inulin extraction. Therefore, we have decided to utilize qualitative analysis. 

Inulin is found in abundance, impacting extract clarity and interfering with result interpretation. As ethanol was used to prepare root extracts, inulin precipitated due to the solvent’s impact on inulin precipitation [50]. This precipitate, in some instances, interfered with result determination in a spectrophotometer. Consequently, it was necessary to filter the resulting extracts to prevent interference from the precipitate. However, filtration might have led to a reduction in the concentration of beneficial chemicals in the extracts, as some of them could have been retained on the filter. Although, inulin was detected in filtered analyte by LC-MS. 

The total phenolic content (TPC) of plants varied significantly depending on the plant, collection location, and extract type. It was observed that there was a statistically significant difference between plants collected in different places, indicating that weather and other conditions impact the content of beneficial substances in plants.

Tannin content was also examined. Tannins are polyphenolic biomolecules found in various natural sources such as nuts, berries, spices, and herbs [51,52]. Their quantification is crucial due to their distinct biological activities and potential health benefits compared to other chemical compounds, including phenolic compounds [53]. Studies highlight that tannins and their metabolites have not only antiviral, anti-inflammatory, and anticancer properties but also antioxidant properties [54,55,56]. For instance, gallic acid, a hydrolyzable tannin, exhibits antibacterial [57], anti-inflammatory, and antioxidant [58,59] effects, as well as protects against oxidative stress [60]. Research by Ajebli [61] also supports the antidiabetic properties of tannins. Additionally, research by Antasionasti [62] indicates that reducing tannin content in extracts decreases the levels of other phenolic compounds and antiradical activity. Given the infrequent study of tannin content in the roots of these plants and their properties, it is crucial to analyze them separately from total phenolics. Unfortunately, we could not associate those antidiabetic effects with dandelion roots, as the results of the spectrophotometric analysis were below the limit of detection. However, this could also be explained by the possibility that the extracts might contain inulin sediment (invisible to the naked eye), hindering the objective determination of the results. Therefore, further studies need to be conducted using alternative solvents. On the other hand, the burdock root extract displayed normal results, suggesting the potential presence of a lower amount of inulin or higher levels of tannin in this plant compared to dandelion.

Each studied chemical compound in these roots exhibits varying antidiabetic properties, requiring additional research for a comprehensive understanding. Further experiments and data collection are essential for a more robust analysis, for example, in vivo tests. The results indicate that burdock root extract, at specific doses, possesses antidiabetic effects through hypolipidemic and antioxidant properties, supporting its potential in DM treatment. Similar conclusions have been drawn in other studies [63,64]. For instance, the study by Akram Ahangarpour et al. [63] demonstrated these effects in mice, and this study validates the antidiabetic effect in a living organism. However, further research is still needed to confirm and validate these results.

Biologically active substances of plant origin have various pharmacodynamic effects, including actions that benefit individuals with T2DM and its complications [65,66]. LC-MS detected numerous beneficial chemical compounds in the root extracts. Research on humans has shown that consuming caffeoylquinic acid-rich extracts over the long term lowers blood glucose levels, increases insulin response [67], alleviates hepatic insulin resistance [68], lowers serum lipids, and facilitates weight loss [69]. These findings substantiate the recommendation of caffeoylquinic acids for DM treatment [70]. Extensive research has been conducted on caffeic acid in experimental DM and its associated complications. Caffeic acid demonstrates hypoglycemic effects [71], enhances insulin levels [72], and ameliorates glucose intolerance [73]. Also, caffeic acid and its derivatives serve as antioxidants, controlling pathways related to how the body handles fats and sugars, and also display antidiabetic effects by influencing certain inflammatory substances and genetic factors [74]. Regular consumption of chlorogenic acid has been shown to lower fasting blood glucose levels, enhance glucose tolerance, promote weight loss, and lower blood pressure in individuals with hypertension [75]. Additionally, another study [76] suggests that it can reduce blood sugar levels and enhance kidney function, even in advanced diabetes. Citric acid significantly reduces blood glucose levels and the insulin resistance index, while also enhancing insulin sensitivity [77]. Malic acid can increase antioxidant activity [78] and is used to treat the consequences of DM, such as xerostomia [79]. Phenylalanine shows antidiabetic effects by enhancing glucose uptake [80]. Oleanolic acid has been shown to enhance insulin response, preserve pancreatic beta cell function, and offer protection against DM complications [81]. Protocatechuic acid demonstrates a potential antihyperglycemic effect comparable to that of glibenclamide [82]. One of the crucial phytochemicals is amino acids, which have significant involvement in numerous metabolic processes [83]. However, one study revealed that the risk of developing DM rose with a higher intake of dietary amino acids, though this trend was not statistically significant for all amino acids [84]. Therefore, it is important to monitor the types and levels of amino acids in plant extracts. While certain compounds lacked confirmed antidiabetic activity and conflicting study data exist, the discovery of numerous compounds with documented benefits for DM supports the potential of these plants to be explored and developed as antioxidant-rich foods or medications with blood sugar-lowering properties.

Examining other studies, an antidiabetic effect was also observed; however, each study presented different results. For example, a study conducted earlier [85] compared the antioxidant activity of dandelion leaves and roots and reported comparisons with the general agreement of higher antioxidant capacity in leaves over roots. In this study, the antioxidant activity of the root extract, expressed as IC50, was 12.6 mg/L, which is lower than that observed in our study for LE (the lower the IC50, the higher the antioxidant activity). This suggests that either external factors during plant growth or shortcomings in conducting experiments influenced the results. In our study, dandelion root, despite its diverse compounds affecting metabolism, digestion, and blood pressure regulation [86], is recommended only in complementary treatment therapy, since its antidiabetic properties are less than those of burdock. However, considering that the effects are likely to be less pronounced than in burdock root, a combined study of both plants in powder form could reveal synergistic effects. 

This study provides valuable insights into the chemical composition and potential therapeutic properties of dandelion and burdock roots. The findings underline the need for further research to unravel the precise mechanisms underlying the reported effects, fostering a better understanding of these plant-derived compounds in the context of DM and related metabolic disorders. It is essential not only to examine the presence of other chemicals in the roots but also to replicate the tests using different solvents, as this could potentially alter the results. It is just as crucial to conduct in vivo tests to comprehend how these plants behave in a living organism.

## 4. Materials and Methods

### 4.1. Chemicals and Reagents

Distilled and purified water (hereafter referred to as water), ethyl alcohol, gallic acid (Acros Organics, Geel, Belgium), Folin–Ciocalteu reagent (Fisher Scientific, Loughborough, UK), sodium carbonate (Honeywell, Charlotte, NC, USA), phosphomolybdic tungstic reagent R, hide powder CRS, pyrogallol, thymol, α-naphthol, sulfuric acid (Fisher Scientific, Loughborough, UK), iodine solution, lead acetate, resorcinol, hydrochloric acid (Fisher Scientific, Loughborough, UK), dimethyl sulfoxide (DMSO) (Sigma-Aldrich, St. Louis, MO, USA), methanol (Sigma-Aldrich, St. Louis, MO, USA), 2,2-diphenyl-1-picrylhydrazyl (DPPH) (Alfa Aesar, Kandel, Germany), Trolox (Acros Organics, Geel, Belgium), sodium phosphate dibasic (Honeywell, Charlotte, NC, USA), potassium sodium tartrate tetrahydrate (Sigma-Aldrich, St. Louis, MO, USA), sodium chloride (Fisher Scientific, Loughborough, UK), α-amylase from porcine pancreas (Sigma-Aldrich, St. Louis, MO, USA), potato starch, 3,5-dinitrosalicylic acid (Sigma-Aldrich, St. Louis, MO, USA), acarbose (Tokyo Chemical Industry CO., LTD, Tokyo, Japan), HPLC-grade methanol (Honeywell, CHROMASOLV, Seelze, Germany), reagent grade formic acid (Assay Ph.Eur ≥ 98%; Sigma-Aldrich, Darmstadt, Germany), ultrapure water Type 1 (prepared using Stackpure purification system; OmniaTap 6, Niederahr, Germany). All solvents used were analytical or HPLC grade.

### 4.2. Plant Material

Dandelion *(Taraxacum officinale*) and burdock (*Arctium lappa*) roots were hand-collected in late autumn from two rural regions in Latvia, encompassing both forests and fields, in 2023. Dandelion was harvested in the “Vecpiebalga” region (57°03′43″ N 25°48′39″ E) and in the inhabited place “Kaļķis,” Jelgava region (56°50′04″ N 23°33′10″ E). Burdock, on the other hand, was harvested near the city of “Viļani” in Rezekne region (56°33′09″ N 26°55′29″ E) and in the inhabited place “Būdiņas,” Jelgava region (56°42′24″ N 23°29′52″ E). All samples were kept with plant voucher codes *BV 2023, BB 2023, DK 2023, DV 2023* at Rīga Stradiņš University pharmacy department in internal herbarium collection.

The root system was lifted out along with the sod of the earth, carefully separated, and, leaving a few centimeters in length, the leaves were trimmed. Subsequently, the roots were placed in a basket, washed under running water, and any unnecessary parts, such as damaged areas or remnants of the stem, were removed. After all the preparations, following the general guidelines outlined by the World Health Organization [87], the roots were dried using artificial heat in an oven until the roots broke when bent. The dried roots were then stored in paper bags in the shade. Finally, the dried roots were ground into a powder.

### 4.3. Preparation of Ethyl Alcohol and Lyophilizate Extracts

**Scheme 1:**
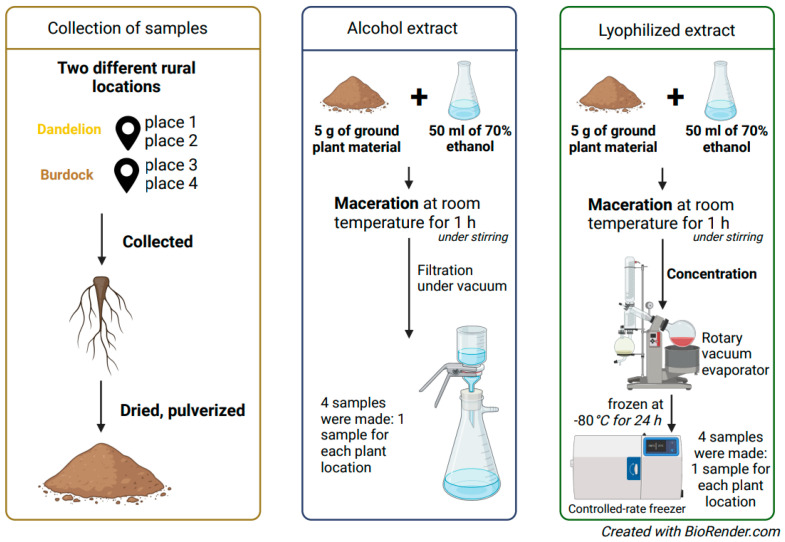
Preparation of ethyl alcohol and lyophilizate extracts.

The extraction was based on the method described by Ma et al. [88] with some modifications. For the preparation of ethyl alcohol extract (AE), 5 g of ground plant material was macerated in 50 mL of 70% ethyl alcohol at room temperature under stirring conditions for 1 h (based on results between article [89] and articles [90,91,92]), followed by filtration under vacuum using filter paper. Lyophilizate extract (LE) was then derived from the already prepared 70% ethyl alcohol extract. The alcohol extract was concentrated using a rotary vacuum evaporator (Model: RV 3 ECO S099) at 37 °C, stored at −80 °C for 24 h, and freeze-dried. A schematic description of the method is shown in Scheme 1.

To ensure uniform conditions for comparison between alcohol and lyophilizate extracts, a larger quantity of alcohol extract was initially prepared. Half was utilized for analyzing the alcohol extract, while the remaining half was utilized for preparing the lyophilized extract.

### 4.4. Extract Analysis

#### 4.4.1. Identification of Inulin

The inulin content was determined using the qualitative reaction described in the State Pharmacopoeia of the Russian Federation XIV edition [93]. For the burdock root, the presence of orange–red coloring (indicating the presence of inulin) should be noted when applying 0.1 mL of a 20% thymol alcohol solution and 0.05 mL of concentrated sulfuric acid to the analyzed powder (ground plant material). For the dandelion root, the presence of a purple–pink color (indicating inulin) should be noted when applying a 20% α-naphthol alcohol solution and concentrated sulfuric acid to the ground plant material. Conversely, the absence of blue staining (indicating the absence of starch) should not be observed in both cases when applying 0.1 mL of iodine solution to the analyzed powder.

#### 4.4.2. Determination of Total Phenolic Content (TPC)

The total phenolic content (TPC) was quantified using the Folin–Ciocalteu colorimetric method [94] with some modifications. For AE analysis, 1 mL of AE was added to 49 mL of water. Then, 1 mL of this dilution was mixed with 5 mL 10% Folin–Ciocalteu reagent and 4 mL 7.5% Na_2_CO_3_ solution. For LE analysis, 0.2 g of LE was diluted in 20 mL 70% ethyl alcohol. A 1 mL amount of this dilution was added to 49 mL water, and then 1 mL of this solution was mixed with 5 mL 10% Folin–Ciocalteu reagent and 4 mL 7.5% Na_2_CO_3_ solution. Both AE and LE samples were incubated for 30 min at room temperature in the dark. Then, absorption was measured at 765 nm using a UV/VIS spectrophotometer (Mettler-Toledo, LabX™, Greifensee, Switzerland). The calibration curve was obtained by combining 1 mL of gallic acid with 5 mL of 10% Folin–Ciocalteu reagent and 4 mL of 7.5% Na_2_CO_3_ solution.

TPC content was expressed as mg of gallic acid equivalent per gram dry material (mg GAE/g DM) and calculated using the expression:

C (mg GAE/g DM) = a × y × (V/m),

where a—extract dilution (times), m—mass of the sample (g), V—ethyl alcohol volume (mL), y—concentration of total phenolic compounds obtained from calibration curve (mg/mL).

#### 4.4.3. Determination of Tannin Level

The tannin level in extracts was determined using the European Pharmacopoeia 8th edition method [95]. For AE analysis, 2 mL of AE was diluted to 250 mL with water. The solution was filtrated under vacuum (with filter paper), and the first 50 mL of filtrate was discarded. A 5 mL amount of the remaining filtrate was diluted to 25 mL with water (action X). Then, 2 mL of this solution was mixed with 1.0 mL of phosphomolybdic tungstic reagent R, and 10.0 mL of water, and diluted to 25.0 mL with a 290 g/L solution of Na_2_CO_3_. After 30 min of incubation in the dark, the absorbance was measured at 760 nm (action Y), using a UV/VIS spectrophotometer (Mettler-Toledo GmbH UV7).

Polyphenols not adsorbed by hide powder CRS were also determined. To 10 mL of the filtrate, 0.10 g of hide powder CRS was added, shaken for 1 h, and filtrated under vacuum. Then, the events from action X to action Y were repeated. For LE analysis, 0.2 g of LE was diluted with water to 250 mL, filtrated, and the first 50 mL was discarded. The process was then repeated as it was for the AE. A standard was prepared by dissolving 50 mg of pyrogallol in water (to 100 mL). A 5 mL amount of this solution was diluted with water to 100 mL. Then, the events after action X and till action Y were repeated.

The tannin content was calculated as a percentage of tannins expressed as pyrogallol in dry material, using the expression:

Tannin content % = [62.5(A1 − A2) m2]/[A3 × m1],

where m1—mass of the sample to be examined (g), m2—mass of pyrogallol (g), A1—absorbance of polyphenols, A2—absorbance of polyphenols that are not adsorbed by hide powder, A3—absorbance of the standard.

#### 4.4.4. Determination of Total Polysaccharide Content (TP)

The total polysaccharide content (TP) in terms of fructose was quantified using the method described in the State Pharmacopoeia of the Russian Federation XIV edition [93]. For the analysis, 1.0 g of ground plant material was mixed with 60 mL of water and heated for 30 min. The extract was then cooled to room temperature and filtrated under vacuum, avoiding the ingress of plant material on the paper filter. The extraction process was repeated thrice, with the last two times using 30 mL of water for 30 min and 15 min, respectively. 

Afterward, the ground plant material was moved to a filter paper and washed with 10 mL of water. To the resulting extract, 2 mL of a 10% lead acetate solution was added and left for 10 min. Subsequently, 2 mL of a 5% sodium phosphate disubstituted solution was added. This mixture was left for 5 min, and water was added to the mark of 200 mL. The entire solution was filtrated under vacuum through a paper filter, discarding the first 15 mL (solution A). A 5 mL amount of solution A was diluted with water to 100 mL and mixed (solution B). Then, 5 mL of a 0.1% resorcinol alcohol solution was combined with 5 mL of solution B and diluted with a 30% hydrochloric acid solution to 25 mL (solution C). For the reference solution, 5 mL of a 0.1% resorcinol alcohol solution was combined with 5 mL of water and diluted with a 30% hydrochloric acid solution to 25 mL. Both the reference solution and solution C were heated in a water bath for 20 min (80 °C). Subsequently, the optical density of both solutions (reference and C) was measured at 482 nm using a UV/VIS spectrophotometer (Mettler-Toledo GmbH UV7). 

TP in terms of fructose was calculated according to the formula:

X (%) = [A × 200 × 100 × 25 × 100]/[A1%1 cm × a × 5 × 5 × (100 − W)],

where A1%1 cm—specific absorption coefficient of the reaction products of fructose with resorcinol (equal to 298), A—optical density of solution B, a—mass of the sample (g), W—moisture content (%).

#### 4.4.5. Determination of Antioxidant Activities of Extracts by Using DPPH (2,2-diphenyl-1-picrylhydrazyl) Assay

The scavenging rate of the DPPH radical was measured according to the procedure reported by Muniandy P. et al. [96] with slight modifications. The ethyl alcohol extract (EA) was prepared in advance, while 0.3 g of lyophilizate extract (LE) had to be dissolved in 5 mL of dimethyl sulfoxide (DMSO). For burdock analysis, 1, 2, 5, 10, and 20 μL of extract were mixed with 29, 28, 25, 20, and 10 μL of DMSO, respectively. For dandelion analysis, 10, 30, 50, 70, and 90 μL of extract were mixed with 90, 70, 50, 30, and 10 μL of DMSO, respectively.

Subsequently, 3 mL of DPPH (0.0118 g of DPPH in 300 mL methanol) was added to a ready-to-use solution of extracts (30 μL for burdock and 100 μL for dandelion), followed by incubation in the dark at room temperature for 15 min. Absorbance was measured at 515 nm against methanol, using a UV/VIS spectrophotometer (Mettler-Toledo GmbH UV7). The control was prepared as a mixture of 30 μL of DMSO and 3 mL of DPPH. A standard Trolox solution with DMSO was used for calibration.

The radical scavenging activity was calculated as follows:

Scavenging activity (%) = [(A1 − A0)/A1] × 100,

where A1—absorbance of control, A0—absorbance of the sample. The antioxidant activity at the end was expressed as IC50 (mg/L).

#### 4.4.6. Hypoglycemic Properties of the Extracts Based on α-Amylase Activity

The ethyl alcohol extract (AE) was diluted to obtain solutions with concentrations of 1, 2, 4, 8, 16, and 20 mg/mL, each with a total volume of 10 mL. Simultaneously, a 32 mg/ mL solution was prepared for the lyophilizate extract (LE), which was then further diluted to concentrations of 16, 8, 4, and 2 mg/mL. The method was adapted from Satvir Sekhon-Loodu [97] with modifications.

To carry out the assay, 200 μL of the extract with a specific concentration was mixed with 200 μL of 70% ethyl alcohol and 200 μL of a solution containing 0.5 mg/mL α-amylase in 0.02 M phosphate buffer (pH 6.9). After incubation at room temperature for 10 min, 200 μL of 1% starch solution was added, and the samples were incubated again for 10 min at the same temperature. Subsequently, 400 μL of the dinitrosalicylic acid color reagent was added, and the samples were placed in a boiling water bath for 5 min and then cooled to room temperature. Finally, 500 μL of this solution was diluted with 2.5 mL of water, and absorbance was measured at 540 nm against the extract solution (100 μL of extract and 2.5 mL of water), using a UV/VIS spectrophotometer (Mettler-Toledo GmbH UV7). 

For comparison, acarbose was used as a known α-amylase inhibitor at concentrations ranging from 1 mg/mL to 20 mg/mL. The control without any inhibitor represented 100% enzyme activity.

The percentage inhibition of the sample was calculated using the formula:

Inhibition (%) = 100 × (Ac − As)/(Ac),

where As—absorbance of the sample, Ac—absorbance of the control. The results were expressed as IC50 (mg/mL).

### 4.5. Qualitative Analysis of Extracts by Liquid Chromatography–Mass Spectrometry (LC-MS)

The method was based on the method described by Ali et al. [98] and Thomas et al. [99] with some modifications. EA was prepared in advance (1 mL was used), while 2 mg of LE had to be dissolved in 1 mL of methanol. The UHPLC-HRMS analyses were carried out using Vanquish Flex UHPLC system (ThermoFisher Scientific, Waltham, MA, USA) consisting of a Vanquish Binary Pump F and Vanquish Split Sampler FT. ACQUITY UPLC HSS T3 column (2.1 × 50 mm, 1.8 μm; Waters, Ireland) was used for chromatographic separation of the compounds. Mobile phase A consisted of 0.1% formic acid in ultrapure water and mobile phase B contained 0.1% formic acid in methanol. The flow rate of the mobile phase was set at 0.3 mL/min and the column temperature was maintained at 30 °C. The gradient program was set as follows: 0 min, 5% B; 1.0 min, 5% B; 15.0 min, 30% B; 20.0 min, 50% B; 25.0 min, 70% B; 26.0 min, 95% B; 28.0 min, 95% B; 29.0 min, 5% B; and 30.0 min, 5% B. Equilibration time was 3 min. The injection volume was 4 μL. Mass spectrometric analysis was performed using Orbitrap Exploris 120 mass spectrometer (ThermoFisher Scientific, Dreieich, Germany) equipped with a heated electrospray ionization (HESI-II) probe (ThermoScientific). The equipment was operated in negative and positive ion modes within the *m*/*z* range from 100 to 1500. The mass spectrometer parameters were as follows: spray voltage 3.5 kV (+) and 2.5 kV (−); sheath gas flow rate 50; auxiliary gas flow rate 10; capillary temperature 325 °C; probe heater temperature 350 °C; S-lens RF level 70; scan mode: full MS (resolution 60,000), and for ddMS2 (15,000), collision energy was normalized and HDC collision energy set at 30%. Data were processed by Xcalibur 4.6 (ThermoScientific, Waltham, MA, USA) instrument control/data handling software. Metabolite profiling using TraceFinder 5.1 software (ThermoScientific, Waltham, MA, USA) was applied to the UHPLC–HRMS raw files of the studied extracts. Based on a variety of literature sources, spectral databases mzCloud, PubChem, FoodDB, and KNApSAcK, an LC–MS library of 250 compounds was created and used for the identification of individual components.

### 4.6. Statistical Analysis

The data are presented as the means ± standard error (SE) of three independent results or as only one result. Descriptive statistics, including one-way ANOVA and Mann–Whitney U test analysis, were performed and analyzed using the IBM^®^ SPSS^®^ Statistics software platform (Version 1.0.28.0, Armonk, NY, USA: IBM Corp.). Significant differences between groups were determined at a *p*-value < 0.05 in all cases.

## 5. Conclusions

In conclusion, the comparative analysis of dandelion (*Taraxacum officinale*) and burdock (*Arctium lappa*) roots has provided insights into their chemical composition and potential therapeutic properties. The study indicates that burdock root exhibited overall better results than dandelion root. However, recommending these plants definitively for diabetes management is currently challenging without thorough comparison with other plants and in vivo studies. 

Nevertheless, the present findings lay the groundwork for future research, highlighting the importance of in vivo studies to determine the safety and efficacy of dandelion and burdock roots as potential interventions for managing diabetes. Further investigations will be essential to comprehensively assess their antidiabetic properties and explore their potential as botanical therapeutics.

## Figures and Tables

**Table 1:** Total phenolic content (TPC) of ethyl alcohol (EA) and lyophilizate (LE) plant extracts.

Plant Sample	TPC (mg GSE/g of EA Extract), ± SE	TPC (mg GSE/g of LE Extract), ± SE
Burdock from “Viļani” (BV)	21.23 ± 0.07 ^a^	100.97 ± 0.49 ^b^
Burdock from “Būdiņas” (BB)	14.36 ± 0.04 ^a^	69.73 ± 0.39 ^b^
Dandelion from “Kaļķis” (DK)	4.61 ± 0.03 ^a^	27.72 ± 0.57 ^b^
Dandelion from “Vecpiebalga” (DV)	4.51 ± 0.03 ^a^	26.35 ± 0.25 ^b^

Within columns, values with the same letter are not significantly different at the *p* > 0.05 level according to ANOVA.

**Table 2:** IC50 value of DPPH radical scavenging activity (mg/L) of ethyl alcohol (EA) and lyophilizate (LE) plant extracts.

Plant Sample	DPPH IC50 (mg/L) for AE	DPPH IC50 (mg/L) for LE	DPPH IC50 (mg/L) for Trolox
Burdock from “Viļani” (BV)	27.95 ^a^	1.33 ^b^	4.62
Burdock from “Būdiņas” (BB)	25.89 ^a^	0.77 ^b^	
Dandelion from “Kaļķis” (DK)	236.32 ^a^	9.52 ^b^	
Dandelion from “Vecpiebalga” (DV)	150.54 ^a^	7.00 ^b^	

Within columns, values with the same letter are significantly different at the *p* < 0.05 level according to ANOVA.

**Table 3:** The IC50 inhibitory values of α-amylase activity of ethyl alcohol (EA) and lyophilizate (LE) plant extracts.

Plant Sample	IC50 (mg/mL) for AE	IC50 (mg/mL) for LE	IC50 (mg/mL) for Acarbose
Burdock from “Viļani” (BV)	135.09 ^a^	79.18 ^b^	18.86
Burdock from “Būdiņas” (BB)	106.67 ^a^	57.94 ^b^	
Dandelion from “Kaļķis” (DK)	205.35 ^a^	142.07 ^b^	
Dandelion from “Vecpiebalga” (DV)	450.11 ^a^	186.43 ^b^	

Within columns, values with the same letter are significantly different at the *p* < 0.05 level according to ANOVA.

**Table 4:** Tentative identification of the chemical constituents of burdock and dandelion extracts by UHPLC-Q-Exactive-MS/MS under positive ionization.

No.	tR (min)	Tentative Compound	Class/Type	Proposed Formula	Ion Mode	Measured *m*/*z*	Error (ppm)	MS/MS
1	0.52	L-Histidine	Amino acid	C_6_H_9_N_3_O_2_	[M+H]^+^	156.08	0.21	138.05; 110.02; 84.04; 73.03
2	0.52	L-Lysine	Amino acid	C_6_H_14_N_2_O_2_	[M+H]^+^	147.11	0.26	130.05; 101.07; 84.05
3	0.53	Arginine	Amino acid	C_6_H_14_N_4_O_2_	[M+H]^+^	175.12	−0.65	158.09; 130.09; 116.07; 70.07
4	0.71	(+)-Valine	Amino acid	C_5_H_11_NO_2_	[M+H]^+^	118.09	−0.08	72.08
5	0.94	L-Tyrosine	Amino acid	C_9_H_11_NO_3_	[M+H]^+^	182.08	−0.13	165.05; 147.04; 136.08; 123.04
6	0.99	L-Leucine/Isoleucine	Amino acid	C_6_H_13_NO_2_	[M+H]^+^	132.10	−0.09	116.03; 86.09; 69.07
7	1.59	(±)-Phenylalanine	Amino acid	C_9_H_11_NO_2_	[M+H]^+^	166.09	−0.20	120.09
8	3.63	L-Tryptophan	Amino acid	C_11_H_12_N_2_O_2_	[M+H]^+^	205.10	−0.21	188.07; 87.04
9	1.94	Pantothenic acid	Amino acid and derivatives	C_9_H_17_NO_5_	[M+H]^+^	220.12	0.00	202.11; 184.10; 90.06
10	0.62	Caffeic acid	Phenylic acid	C_9_H_8_O_4_	[M+H]^+^	181.05	0.20	163.04
11	5.28	Neochlorogenic acid	Phenylic acid	C_16_H_18_O_9_	[M+H]^+^	355.10	−0.44	163.04
12	5.63	Chlorogenic acid	Phenylic acid	C_16_H_18_O_9_	[M+H]^+^	355.10	−0.44	163.04
13	11.20	3,4-Dicaffeoylquinic acid	Phenylic acid	C_25_H_24_O_12_	[M+H]^+^	517.13	−0.39	163.04
14	11.54	3,5-Dicaffeoylquinic acid	Phenylic acid	C_25_H_24_O_12_	[M+H]^+^	517.13	−0.39	163.04
15	12.37	4,5-Dicaffeoylquinic acid	Phenylic acid	C_25_H_24_O_12_	[M+H]^+^	517.13	−0.15	163.04
16	0.62	Trigonelline	Alkaloids	C_7_H_7_NO_2_	[M+H]^+^	138.05	0.24	128.11
17	11.90	Eremanthin	Guaianolides	C_15_H_18_O_2_	[M+H]^+^	231.14	−0.22	213.13; 185.13; 175.07
18	19.62	Campholenic aldehyde	Monocyclic monoterpenoids	C_10_H_16_O	[M+H]^+^	153.13	−0.06	135.12; 109.10; 107.09; 97.06
19	21.50	Arctinone A	Oligothiophenes	C_13_H_10_O_2_S_2_	[M+H]^+^	263.02	−0.11	263.02; 245.01; 217.02; 205.02

**Table 5:** Tentative identification of the chemical constituents of burdock and dandelion extracts by UHPLC-Q-Exactive-MS/MS under negative ionization.

No.	tR (min)	Tentative Compound	Class/Type	Proposed Formula	Ion Mode	Measured *m*/*z*	Error (ppm)	MS/MS
1	0.59	Disaccharide	Carbohydrates	C_12_H_22_O_11_	[M-H]^−^	341.11	−0.81	179.05; 89.02; 59.01
2	0.68	Malic acid	Hydroxy acid	C_4_H_6_O_5_	[M-H]^−^	133.01	−0.33	133.01; 115.00; 71.01
3	1.51	Galloyl glucose	Tannins	C_13_H_16_O_10_	[M-H]^−^	331.07	0.48	313.06; 168.01; 125.02
4	2.08	Protocatechuic acid 4-glucoside	Phenolic glycosides	C_13_H_16_O_9_	[M-H]^−^	315.07	0.14	315.07; 153.02; 152.01; 109.03. 108.02
5	3.27	3-Methoxy-4-hydroxyphenylglycol glucuronide	Phenolic glycosides	C_15_H_20_O_10_	[M-H]^−^	359.10	−0.97	197.05; 182.02; 153.06; 138.03
6	5.28	Neochlorogenic acid	Phenylic acid	C_16_H_18_O_9_	[M-H]^−^	353.09	−0.98	191.05
7	5.40	n-Caffeoylquinic acid	Phenylic acid	C_16_H_18_O_9_	[M-H]^−^	353.09	−1.07	191.05
8	5.76	Chlorogenic acid	Phenylic acid	C_16_H_18_O_9_	[M-H]^−^	353.09	−0.98	191.05
9	8.38	p-coumaric acid	Phenylic acid	C_9_H_8_O_3_	[M-H]^−^	163.04	0.00	119.05
10	11.25	3,4-Dicaffeoylquinic acid	Phenylic acid	C_25_H_24_O_12_	[M-H]^−^	515.12	−0.46	353.09; 191.06
11	11.57	3,5-Dicaffeoylquinic acid	Phenylic acid	C_25_H_24_O_12_	[M-H]^−^	515.12	−0.70	353.09; 191.06
12	12.42	4,5-Dicaffeoylquinic acid	Phenylic acid	C_25_H_24_O_12_	[M-H]^−^	515.12	−0.70	353.09; 191.06
13	15.16	Caffeic acid ethyl ester	Coumaric acids and derivatives	C_11_H_12_O_4_	[M-H]^−^	207.07	−0.79	208.07; 179.03; 161.02
14	6.46	Protocatechuic acid	Benzoic acid and derivatives	C_7_H_6_O_4_	[M-H]^−^	153.02	0.12	153.02; 135.01; 109.03
15	4.28	Salicylic acid	Benzoic acid and derivatives	C_7_H_6_O_3_	[M-H]^−^	137.02	−0.04	93.03
16	4.76	Salicylic acid glucoside	Benzoic acid and derivatives	C_13_H_16_O_8_	[M-H]^−^	299.08	0.00	137.02; 93.03
17	27.85	Oleanolic acid	Pentacyclic triterpene	C_30_H_48_O_3_	[M-H]^−^	455.35	−0.43	455.35

**Table 6:** Presence of chemical constituents of burdock and dandelion extracts by UHPLC-Q-Exactive-MS/MS under positive ionization.

No.	Tentative Compound	Class/Type	Plant Sample							
			LE of BB	AE of BB	LE of BV	AE of BV	LE of DK	AE of DK	LE of DV	AE of DV
1	L-histidine	Amino acid	X	X	X	X	n.d.	X	n.d.	n.d.
2	L-lysine	Amino acid	X	X	X	X	n.d.	n.d.	n.d.	X
3	Arginine	Amino acid	X	X	X	X	X	X	X	X
4	Caffeic acid	Phenylic acid	X	X	n.d.	n.d.	n.d.	n.d.	n.d.	n.d.
5	Trigonelline	Alkaloids	X	X	X	n.d.	n.d.	n.d.	n.d.	n.d.
6	(+)-Valine	Amino acid	X	X	X	X	X	X	X	X
7	L-tyrosine	Amino acid	X	X	X	X	n.d.	X	n.d.	n.d.
8	L-leucine/isoleucine	Amino acid	X	X	X	X	n.d.	X	X	X
9	(±)-Phenylalanine	Amino acid	X	X	X	X	X	X	X	X
10	Pantothenic acid	Amino acid and derivatives	n.d.	n.d.	n.d.	X	n.d.	n.d.	n.d.	X
11	L-tryptophan	Amino acid	X	X	X	X	n.d.	X	X	X
12	Neochlorogenic acid	Phenylic acid	X	X	X	X	n.d.	X	X	X
13	Chlorogenic acid	Phenylic acid	X	X	X	X	n.d.	X	X	X
14	3,4-Dicaffeoylquinic acid	Phenylic acid	X	n.d.	X	X	n.d.	n.d.	n.d.	n.d.
15	3,5-Dicaffeoylquinic acid	Phenylic acid	X	X	X	X	n.d.	X	n.d.	X
16	Eremanthin	Guaianolides	n.d.	n.d.	n.d.	n.d.	n.d.	n.d.	n.d.	X
17	4,5-Dicaffeoylquinic acid	Phenylic acid	X	X	X	X	n.d.	X	n.d.	n.d.
18	Campholenic aldehyde	Monocyclic monoterpenoids	n.d.	n.d.	n.d.	n.d.	n.d.	n.d.	n.d.	X
19	Arctinone A	Oligothiophenes	n.d.	X	n.d.	X	n.d.	n.d.	n.d.	n.d.

n.d. = not detected. Seventy percent ethyl alcohol extract (AE), lyophilizate extracts (LE). Burdock from “Viļani” (BV), burdock from “Būdiņas” (BB), dandelion from “Kaļķis” (DK), dandelion from “Vecpiebalga” (DV).

**Table 7:** Presence of chemical constituents of burdock and dandelion extracts by UHPLC-Q-Exactive-MS/MS under negative ionization.

No.	Tentative Compound	Class/Type	Plant Sample							
			LE of BB	AE of BB	LE of BV	AE of BV	LE of DK	AE of DK	LE of DV	AE of DV
1	Disaccharide	Carbohydrates	X	X	X	X	X	X	X	X
2	Malic acid	Hydroxy acid	n.d.	X	X	X	X	X	X	X
3	Galloyl glucose	Tannins	n.d.	X	n.d.	X	n.d.	X	n.d.	X
4	Protocatechuic acid 4-glucoside	Phenolic glycosides	n.d.	X	n.d.	X	X	X	X	X
5	3-Methoxy-4-hydroxyphenylglycol glucuronide	Phenolic glycosides	n.d.	X	X	X	X	X	n.d.	X
6	Salicylic acid	Benzoic acid and derivatives	n.d.	X	n.d.	X	X	X	X	X
7	Salicylic acid glucoside	Benzoic acid and derivatives	n.d.	n.d.	n.d.	n.d.	n.d.	X	n.d.	X
8	Neochlorogenic acid	Phenylic acid	X	X	X	X	X	X	X	X
9	n-Caffeoylquinic acid	Phenylic acid	X	X	X	X	X	X	X	X
10	Chlorogenic acid	Phenylic acid	X	X	X	X	X	X	X	X
11	Protocatechuic acid	Benzoic acid and derivatives	n.d.	n.d.	n.d.	n.d.	n.d.	X	n.d.	n.d.
12	p-coumaric acid	Phenylic acid	n.d.	X	n.d.	X	n.d.	n.d.	n.d.	n.d.
13	3,4-Dicaffeoylquinic acid	Phenylic acid	X	X	X	X	n.d.	n.d.	X	X
14	3,5-Dicaffeoylquinic acid	Phenylic acid	X	X	X	X	X	X	X	X
15	4,5-Dicaffeoylquinic acid	Phenylic acid	X	X	X	X	X	X	X	X
16	Caffeic acid ethyl ester	Coumaric acids and derivatives	n.d.	n.d.	n.d.	X	n.d.	n.d.	n.d.	n.d.
17	Oleanolic acid	Pentacyclic triterpene	X	X	n.d.	X	n.d.	n.d.	n.d.	n.d.

n.d. = not detected. Seventy percent ethyl alcohol extract (AE), lyophilizate extracts (LE). Burdock from “Viļani” (BV), burdock from “Būdiņas” (BB), dandelion from “Kaļķis” (DK), dandelion from “Vecpiebalga” (DV).

## Data Availability

Data are contained within the article.

## References

[1] Ojo O.A., Ibrahim H.S., Rotimi D.E., Ogunlakin A.D., Ojo A.B. (2023). Diabetes mellitus: From molecular mechanism to pathophysiology and pharmacology. Med. Nov. Technol. Devices.

[2] International Diabetes Federation (2021). IDF Diabetes Atlas.

[3] Cho N.H., Shaw J.E., Karuranga S., Huang Y., Fernandes da Rocha J.D., Ohlrogge A.W., Malanda B. (2018). Global estimates of diabetes prevalence for 2017 and projections for 2045. Diabetes Research and Clinical Practice. IDF Diabetes Atlas.

[4] Galicia-Garcia U., Benito-Vicente A., Jebari S., Larrea-Sebal A., Siddiqi H., Uribe K.B., Ostolaza H., Martín C. (2020). Pathophysiology of Type 2 Diabetes Mellitus. Int. J. Mol. Sci..

[5] Park S., Lee G., Lee H., Hoang T., Chae H. (2020). Glucose-lowering effect of Gryllus bimaculatus powder on streptozotocin-induced diabetes through the AKT/mTOR pathway. Food Sci. Nutr..

[6] Antar S.A., Ashour N.A., Sharaky M., Khattab M., Ashour N.A., Zaid R.T., Roh E.J., Elkamhawy A., Al-Karmalawy A.A. (2023). Diabetes mellitus: Classification, mediators, and complications; A gate to identify potential targets for the development of new effective treatments. Biomed. Pharmacother..

[7] Yilmaz O., Ersan Y., Dilek Ozsahin A., Ihsan Ozturk A., Ozkan Y. (2013). Consequences of the Combined α-tocopherol, Ascorbic Acid and α-lipoic Acid on the Glutathione, Cholesterol and Fatty Acid Composition in Muscle and Liver of Diabetic Rats. Iran J. Basic Med. Sci..

[8] Chander A.P., Reddy R.A., Puchchakayala G. (2011). Hypoglycemic and Antidiabetic Activity of Glochidion velutinum on Streptozotocin-Nicotinamide Induced Type 2 Diabetic Rats. Eur. J. BiolSci..

[9] Bindu J., Narendhirakannan R.T. (2019). Role of medicinal plants in the management of diabetes mellitus: A review. 3 Biotech.

[10] Przeor M. (2022). Some Common Medicinal Plants with Antidiabetic Activity, Known and Available in Europe (A Mini-Review). Pharmaceuticals.

[11] Ndip R.N., Tanih N.F., Kuete V. (2013). Antidiabetes Activity of African Medicinal Plants. Medicinal Plant Research in Africa: Pharmacology and Chemistry.

[12] Arya A., Nyamathulla S., Noordin M.I., Mohd M.A. (2012). Antioxidant and Hypoglycemic Activities of Leaf Extracts of Three Popular Terminalia Species. J. Chem..

[13] Lis B., Olas B. (2019). Pro-health activity of dandelion (*Taraxacum officinale* L.) and its food products—History and present. J. Funct. Foods.

[14] Yosri N., Alsharif S.M., Xiao J., Musharraf S.G., Zhao C., Saeed A., Gao R., Said N.S., Di Minno A., Daglia M. (2023). Arctium lappa (Burdock): Insights from ethnopharmacology potential, chemical constituents, clinical studies, pharmacological utility and nanomedicine. Biomed. Pharmacother..

[15] González-Castejón M., Visioli F., Rodriguez-Casado A. (2012). Diverse biological activities of dandelion. Nutr. Rev..

[16] Olennikov D.N., Kashchenko N.I., Chirikova N.K., Koryakina L.P., Vladimirov L.N. (2015). Bitter Gentian Teas: Nutritional and Phytochemical Profiles, Polysaccharide Characterisation and Bioactivity. Molecules.

[17] Monmai C., Park S.H., You S., Park W.J. (2017). Immuno-enhancement effect of polysaccharide extracted from Stichopus japonicus on cyclophosphamide-induced immunosuppression mice. Food Sci. Biotechnol..

[18] Su J., Liu X., Li H., Cheng X., Shi S., Li N., Wu J., Xu Y., Liu R., Tian X. (2020). Hypoglycaemic effect and mechanism of an RG-II type polysaccharide purified from Aconitum coreanum in diet-induced obese mice. Int. J. Biol. Macromol..

[19] Patel D.K., Kumar R., Laloo D., Hemalatha S. (2012). Diabetes mellitus: An overview on its pharmacological aspects and reported medicinal plants having antidiabetic activity. Asian Pac. J. Trop. Biomed..

[20] Artasensi A., Pedretti A., Vistoli G., Fumagalli L. (2020). Type 2 Diabetes Mellitus: A Review of Multi-Target Drugs. Molecules.

[21] Akhtar W., Ali G., Ashraf N., Fatima I., Kayani W.K., Shaheen H., Ghoneim M.M., Abdelgawad M.A., Khames A. (2022). Efficiency of Multiple Extraction Solvents on Antioxidant, Cytotoxic, and Phytotoxic Potential of *Taraxacum officinale* (L.) Weber ex F.H. Wigg. from Poonch Valley, Azad Kashmir, Pakistan. Evid.-Based Complement. Altern. Med..

[22] Moayyed M., Eftekharian A., Fereiduni M., Akbary P. (2023). Research Article: Effect of solvent type on phytochemical properties of burdock (*Arctium lappa*) extract and their effect on some pathogenic bacteria strains in rainbow trout, Oncorhynchus mykiss. IJFS.

[23] Zhang Z., Zhang L., Xu H. (2019). Effect of Astragalus polysaccharide in treatment of diabetes mellitus: A narrative review. J. Tradit. Chin. Med..

[24] Hu F., Li X., Zhao L., Feng S., Wang C. (2010). Antidiabetic properties of purified polysaccharide from Hedysarum polybotrys. Can. J. Physiol. Pharmacol..

[25] Birkeland E., Gharagozlian S., Birkeland K.I., Valeur J., Måge I., Rud I., Aas A.M. (2020). Prebiotic effect of inulin-type fructans on faecal microbiota and short-chain fatty acids in type 2 diabetes: A randomised controlled trial. Eur. J. Nutr..

[26] Muflihah Y.M., Gollavelli G., Ling Y.-C. (2021). Correlation Study of Antioxidant Activity with Phenolic and Flavonoid Compounds in 12 Indonesian Indigenous Herbs. Antioxidants.

[27] Tong Z., He W., Fan X., Guo A. (2022). Biological Function of Plant Tannin and Its Application in Animal Health. Front Vet. Sci..

[28] Alam M.N., Bristi N.J., Rafiquzzaman M. (2013). Review on in vivo and in vitro methods evaluation of antioxidant activity. Saudi Pharm. J..

[29] Shori A.B. (2020). Inclusion of phenolic compounds from different medicinal plants to increase α-amylase inhibition activity and antioxidants in yogurt. J. Taibah Univ. Sci..

[30] Ali H., Houghton P.J., Soumyanath A. (2006). α-Amylase inhibitory activity of some Malaysian plants used to treat diabetes; with particular reference to Phyllanthus amarus. J. Ethnopharmacol..

[31] Zhang P., Li T., Wu X., Nice E.C., Huang C., Zhang Y. (2020). Oxidative stress and diabetes: Antioxidative strategies. Front. Med..

[32] Daou M., Elnaker N.A., Ochsenkühn M.A., Amin S.A., Yousef A.F., Yousef L.F. (2022). In vitro α-glucosidase inhibitory activity of Tamarix nilotica shoot extracts and fractions. PLoS ONE.

[33] Ansari P., Flatt P.R., Harriott P., Abdel-Wahab Y.H.A. (2022). Insulin secretory and antidiabetic actions of Heritiera fomes bark together with isolation of active phytomolecules. PLoS ONE.

[34] Doan H.V., Riyajan S., Iyara R., Chudapongse N. (2018). Antidiabetic activity, glucose uptake stimulation and α-glucosidase inhibitory effect of *Chrysophyllum cainito* L. stem bark extract. BMC Complement. Altern. Med..

[35] Bhatti J.S., Sehrawat A., Mishra J., Sidhu I.S., Navik U., Khullar N., Kumar S., Bhatti G.K., Reddy P.H. (2022). Oxidative stress in the pathophysiology of type 2 diabetes and related complications: Current therapeutics strategies and future perspectives. Free Radic. Biol. Med..

[36] Evans J.L., Goldfine I.D., Maddux B.A., Grodsky G.M. (2002). Oxidative Stress and Stress-Activated Signaling Pathways: A Unifying Hypothesis of Type 2 Diabetes. Endocr. Rev..

[37] Caturano A., D’Angelo M., Mormone A., Russo V., Mollica M.P., Salvatore T., Galiero R., Rinaldi L., Vetrano E., Marfella R. (2023). Oxidative Stress in Type 2 Diabetes: Impacts from Pathogenesis to Lifestyle Modifications. Curr. Issues Mol. Biol..

[38] Munteanu I.G., Apetrei C. (2021). Analytical Methods Used in Determining Antioxidant Activity: A Review. Int. J. Mol. Sci..

[39] Sun Y. (2011). Structure and biological activities of the polysaccharides from the leaves, roots and fruits of Panax ginseng C.A. Meyer: An overview. Carbohydr. Polym..

[40] Ooi V.E., Liu F. (2000). Immunomodulation and anti-cancer activity of polysaccharide-protein complexes. Curr. Med. Chem..

[41] Yu Y., Shen M., Song Q., Xie J. (2018). Biological activities and pharmaceutical applications of polysaccharide from natural resources: A review. Carbohydr. Polym..

[42] Zhang S., Liu X., Yan L., Zhang Q., Zhu J., Huang N., Wang Z. (2015). Chemical compositions and antioxidant activities of polysaccharides from the sporophores and cultured products of Armillaria mellea. Molecules.

[43] Gupta N., Jangid A.K., Pooja D., Kulhari H. (2019). Inulin: A novel and stretchy polysaccharide tool for biomedical and nutritional applications. Int. J. Biol. Macromol..

[44] Wang L., Yang H., Huang H., Zhang C., Zuo H.X., Xu P., Niu Y.M., Wu S.S. (2019). Inulin-type fructans supplementation improves glycemic control for the prediabetes and type 2 diabetes populations: Results from a GRADE-assessed systematic review and dose-response meta-analysis of 33 randomized controlled trials. J. Transl. Med..

[45] Segar H.M., Gani S.A., Khayat M.E., Rahim M.B.H.A. (2023). Antioxidant and Antidiabetic Properties of Pectin Extracted from Pomegranate (*Punica granatum*) Peel. J. Biochem. Microbiol. Biotechnol..

[46] Lu N., Wei J., Gong X., Tang X., Zhang X., Xiang W., Liu S., Luo C., Wang X. (2023). Preventive Effect of Arctium lappa Polysaccharides on Acute Lung Injury through Anti-Inflammatory and Antioxidant Activities. Nutrients.

[47] Li M., Zhang H., Hu X., Liu Y., Liu Y., Song M., Wu R., Wu J. (2022). Isolation of a New Polysaccharide from Dandelion Leaves and Evaluation of Its Antioxidant, Antibacterial, and Anticancer Activities. Molecules.

[48] Escobar-Ledesma F.R., Sánchez-Moreno V.E., Vera E., Ciobotă V., Jentzsch P.V., Jaramillo L.I. (2020). Extraction of Inulin from Andean Plants: An Approach to Non-Traditional Crops of Ecuador. Molecules.

[49] Zeaiter Z., Regonesi M.E., Cavini S., Labra M., Sello G., Di Gennaro P. (2019). Extraction and Characterization of Inulin-Type Fructans from Artichoke Wastes and Their Effect on the Growth of Intestinal Bacteria Associated with Health. BioMed. Res. Int..

[50] Ku Y., Jansen O., Oles C.J., Lazar E.Z., Rader J.I. (2003). Precipitation of inulins and oligoglucoses by ethanol and other solvents. Food Chem..

[51] Puupponen-Pimiä R., Nohynek L., Meier C., Kähkönen M., Heinonen M., Hopia A., Oksman-Caldentey K.M. (2001). Antimicrobial properties of phenolic compounds from berries. J. Appl. Microbiol..

[52] Vattem D.A., Ghaedian R., Shetty K. (2005). Enhancing health benefits of berries through phenolic antioxidant enrichment: Focus on cranberry. Asia Pac. J. Clin. Nutr..

[53] Aba P.E., Asuzu I.U. (2018). Mechanisms of actions of some bioactive anti-diabetic principles from phytochemicals of medicinal plants: A review. Indian J. Nat. Prod. Resour..

[54] Okuda T. (2005). Systematics and Health Effects of Chemically Distinct Tannins in Medicinal Plants. Phytochemistry.

[55] Chung K.T., Wong T.Y., Wei C.I., Huang Y.W., Lin Y. (1998). Tannins and human health: A review. Crit. Rev. Food Sci. Nutr..

[56] Szczurek A. (2021). Perspectives on Tannins. Biomolecules.

[57] Hou A.-J., Liu Y.-Z., Yang H., Lin Z.-W., Sun H.-D. (2000). Hydrolyzable Tannins and Related Polyphenols from Eucalyptus globulus. J. Asian Nat. Prod. Res..

[58] Bai J., Zhang Y., Tang C., Hou Y., Ai X., Chen X., Zhang Y., Wang X., Meng X. (2021). Gallic Acid: Pharmacological Activities and Molecular Mechanisms Involved in Inflammation-Related Diseases. Biomed. Pharmacother..

[59] Singla E., Dharwal V., Naura A.S. (2020). Gallic Acid Protects against the COPD-Linked Lung Inflammation and Emphysema in Mice. Inflamm. Res..

[60] Shree A., Islam J., Vafa A., Mohammad Afzal S., Sultana S. (2020). Gallic Acid Prevents 1, 2-Dimethylhydrazine Induced Colon Inflammation, Toxicity, Mucin Depletion, and Goblet Cell Disintegration. Environ. Toxicol..

[61] Ajebli M., Eddouks M. (2019). The Promising Role of Plant Tannins as Bioactive Antidiabetic Agents. Curr. Med. Chem..

[62] Antasionasti I., Datu O.S., Lestari U.S., Abdullah S.S., Jayanto I. (2021). Correlation Analysis of Antioxidant Activities with Tannin, Total Flavonoid, and Total Phenolic Contents of Nutmeg (Myristica Fragrans Houtt) Fruit Precipitated by Egg White. Borneo J. Pharm..

[63] Ahangarpour A., Heidari H., Oroojan A.A., Mirzavandi F., Nasr Esfehani K., Dehghan Mohammadi Z. (2017). Antidiabetic, hypolipidemic and hepatoprotective effects of Arctium lappa root’s hydro-alcoholic extract on nicotinamide-streptozotocin induced type 2 model of diabetes in male mice. Avicenna J. Phytomed..

[64] Yuan P.-C., Shao T.-L., Han J., Liu C.-Y., Wang G.-D., He S.-G., Xu S.-X., Nian S.-H., Chen K.-S. (2021). Burdock fructooligosaccharide as an α-glucosidase inhibitor and its antidiabetic effect on high-fat diet and streptozotocin-induced diabetic mice. J. Funct. Foods.

[65] Kritsak M., Stechyshyn I., Pavliuk B., Konovalenko S. (2021). Analysis of patients’ rehabilitation results after surgical treatment of diabetes complications. Pol. Merkur. Lek..

[66] Skyler J.S., Bakris G.L., Bonifacio E., Darsow T., Eckel R.H., Groop L., Groop P.-H., Handelsman Y., Insel R.A., Mathieu C. (2017). Differentiation of diabetes by pathophysiology, natural history, and prognosis. Diabetes.

[67] Reis C.E.G., Dórea J.G., da Costa T.H.M. (2018). Effects of coffee consumption on glucose metabolism: A systematic review of clinical trials. J. Tradit. Complement. Med..

[68] Lecoultre V., Carrel G., Egli L., Binnert C., Boss A., MacMillan E.L., Kreis R., Boesch C., Darimont C., Tappy L. (2014). Coffee consumption attenuates short-term fructose-induced liver insulin resistance in healthy men. Am. J. Clin. Nutr..

[69] Martínez-López S., Sarriá B., Mateos R., Bravo-Clemente L. (2019). Moderate consumption of a soluble green/roasted coffee rich in caffeoylquinic acids reduces cardiovascular risk markers: Results from a randomized, cross-over, controlled trial in healthy and hypercholesterolemic subjects. Eur. J. Nutr..

[70] Spínola V., Castilho P.C. (2017). Evaluation of Asteraceae herbal extracts in the management of diabetes and obesity. Contribution of caffeoylquinic acids on the inhibition of digestive enzymes activity and formation of advanced glycation end-products (in vitro). Phytochemistry.

[71] Celik S., Erdogan S., Tuzcu M. (2009). Caffeic acid phenethyl ester (CAPE) exhibits significant potential as an antidiabetic and liver-protective agent in streptozotocin-induced diabetic rats. Pharmacol. Res..

[72] Chao C.Y., Mong M.C., Chan K.C., Yin M.C. (2010). Anti-glycative and anti-inflammatory effects of caffeic acid and ellagic acid in kidney of diabetic mice. Mol. Nutr. Food Res..

[73] Bezerra R.M.N., Veiga L.F., Caetano A.C., Rosalen P.L., Amaral M.E.C., Palanch A.C., de Alencar S.M. (2012). Caffeic acid phenethyl ester reduces the activation of the nuclear factor κB pathway by high-fat diet-induced obesity in mice. Metabolism.

[74] Ganguly R., Singh S.V., Jaiswal K., Kumar R., Pandey A.K. (2023). Modulatory effect of caffeic acid in alleviating diabetes and associated complications. World J. Diabetes.

[75] Yu Y., Zhang Z., Chang C. (2022). Chlorogenic acid intake guidance: Sources, health benefits, and safety. Asia Pac. J. Clin. Nutr..

[76] Jin S., Chang C., Zhang L., Liu Y., Huang X., Chen Z. (2015). Chlorogenic acid improves late diabetes through adiponectin receptor signaling pathways in db/db mice. PLoS ONE.

[77] Yadikar N., Ahmet A., Zhu J., Bao X., Yang X., Han H., Rozi P. (2022). Exploring the mechanism of citric acid for treating glucose metabolism disorder induced by hyperlipidemia. J. Food Biochem..

[78] Li N., Li Q., He X., Gao X., Wu L., Xiao M., Cai W., Liu B., Zeng F. (2022). Antioxidant and anti-aging activities of Laminaria japonica polysaccharide in Caenorhabditis elegans based on metabonomic analysis. Int. J. Biol. Macromol..

[79] Muhamed S.A., Moussa E.M., Aboasy N.K., Gaweesh Y.Y. (2024). Effect of 1% malic acid spray on diabetes mellitus-induced xerostomia: A randomized clinical trial. Oral Dis..

[80] Srinivasan V., Radhakrishnan S., Angayarkanni N., Sulochana K.N. (2019). Antidiabetic effect of free amino acids supplementation in human visceral adipocytes through adiponectin-dependent mechanism. Indian J. Med. Res..

[81] Errichiello F., D’Amato M., Gambuti A., Moio L., Pastore A., Al-Hmadi H., Stornaiuolo M., Serino E., Taglialatela-Scafati O., Forino M. (2023). Oleanolic acid: A promising antidiabetic metabolite detected in Aglianico grape pomace. J. Funct. Foods.

[82] Harini R., Pugalendi K.V. (2010). Antihyperglycemic effect of protocatechuic acid on streptozotocin-diabetic rats. J. Basic Clin. Physiol. Pharmacol..

[83] Comerford K.B., Pasin G. (2016). Emerging Evidence for the Importance of Dietary Protein Source on Glucoregulatory Markers and Type 2 Diabetes: Different Effects of Dairy, Meat, Fish, Egg, and Plant Protein Foods. Nutrients.

[84] Najafi F., Mohseni P., Pasdar Y., Niknam M., Izadi N. (2023). The association between dietary amino acid profile and the risk of type 2 diabetes: Ravansar non-communicable disease cohort study. BMC Public Health.

[85] García-Carrasco B., Fernandez-Dacosta R., Dávalos A., Ordovás J.M., Rodriguez-Casado A. (2015). In vitro Hypolipidemic and Antioxidant Effects of Leaf and Root Extracts of Taraxacum Officinale. Med. Sci..

[86] Kania-Dobrowolska M., Baraniak J. (2022). Dandelion (*Taraxacum officinale* L.) as a Source of Biologically Active Compounds Supporting the Therapy of Co-Existing Diseases in Metabolic Syndrome. Foods.

[87] World Health Organization (2003). WHO Guidelines on Good Agricultural and Collection Practices (GACP) for Medicinal Plants.

[88] Ma X., Wu H., Liu L., Yao Q., Wang S., Zhan R., Xing S., Zhou Y. (2011). Polyphenolic compounds and antioxidant properties in mango fruits. Sci. Hortic..

[89] Maimulyanti A., Prihadi A.R., Mellisani B., Nurhidayati I., Putri FA R., Puspita F., Widarsih R. (2023). Green Extraction Technique to Separate Tannin from Coffee Husk Waste Using Natural Deep Eutectic Solvent (Nades). Rasayan J. Chem..

[90] Ćujić N., Šavikin K., Janković T., Pljevljakušić D., Zdunić G., Ibrić S. (2016). Optimization of polyphenols extraction from dried chokeberry using maceration as traditional technique. Food Chem..

[91] Cacique A.P., Barbosa S., de Pinho G.P., Silvério F.O. (2020). Maceration extraction conditions for determining the phenolic compounds and the antioxidant activity of *Catharanthus roseus* (L.) G. Don. Ciênc. Agrotec..

[92] Kehili M., Sayadi S., Frikha F., Zammel A., Allouche N. (2019). Optimization of lycopene extraction from tomato peels industrial by-product using maceration in refined olive oil. Food Bioprod. Process..

[93] The Russian Federation Ministry of Health State Pharmacopoeia of Russian Federation, XIV and II ed., Volume IV, 2018. Federal Electronic Medical Library. https://femb.ru/record/pharmacopea14.

[94] Kähkönen M.P., Hopia A.I., Vuorela H.J., Rauha J.-P., Pihlaja K., Kujala T.S., Heinonen M. (1999). Antioxidant Activity of Plant Extracts Containing Phenolic Compounds. J. Agric. Food Chem..

[95] European Council, European Directorate for the Quality of Medicines & HealthCare (EDQM) (2013). Tannins in Herbal Drugs. European Pharmacopoeia.

[96] Muniandy P., Shori A.B., Baba A.S. (2016). Influence of green, white and black tea addition on the antioxidant activity of probiotic yogurt during refrigerated storage. Food Packag. Shelf Life.

[97] Sekhon-Loodu S., Rupasinghe H.P.V. (2019). Evaluation of Antioxidant, Antidiabetic and Antiobesity Potential of Selected Traditional Medicinal Plants. Front. Nutr..

[98] Ali A., Bashmil Y.M., Cottrell J.J., Suleria H.A.R., Dunshea F.R. (2021). LC-MS/MS-QTOF Screening and Identification of Phenolic Compounds from Australian Grown Herbs and Their Antioxidant Potential. Antioxidants.

[99] Thomas S.N., French D., Jannetto P.J., Rappold B.A., Clarke W.A. (2022). Liquid chromatography-tandem mass spectrometry for clinical diagnostics. Nat. Rev. Methods Primers.

